# Successful Therapy with Obinutuzumab in a Toddler with Primary Multidrug-Resistant Nephrotic Syndrome

**DOI:** 10.3390/jcm15010060

**Published:** 2025-12-22

**Authors:** Magdalena Drozynska-Duklas, Ilona Zagozdzon, Ilona Chudzik, Irena Balasz-Chmielewska, Anna Kranz, Aleksandra Zurowska

**Affiliations:** Department of Paediatrics, Nephrology and Hypertension, Medical University of Gdansk, 80-210 Gdansk, Poland; ilona.zagozdzon@gumed.edu.pl (I.Z.); ilona.chudzik@gumed.edu.pl (I.C.); ibalasz@gumed.edu.pl (I.B.-C.); anna.kranz@gumed.edu.pl (A.K.); aleksandra.zurowska@gumed.edu.pl (A.Z.)

**Keywords:** multidrug-resistant nephrotic syndrome, children, obinutuzumab

## Abstract

Background/Objectives: Primary steroid-resistant nephrotic syndrome (SRNS) in children is an ominous diagnosis due to limited therapeutic options and poor prognosis. The younger the child, the greater the probability of a genetic etiology which is typically resistant to immunosuppressive therapy. International guidelines recommend genetic testing and a search for rare infectious causes in the youngest age group. When no identifiable etiology is found, an immunologic cause of SRNS is suspected, with few therapeutic options available, which lately have included anti-CD20 therapy for children > 7 years of age. This is the first report on the successful use of obinutuzumab in a very young child with primary SRNS. Methods: Two consecutive doses of obinutuzumab, a humanized anti-CD20 antibody (300 mg/m^2^ BSA), were administered two weeks apart to a 24-month-old boy with severe, complicated SRNS which had been refractory to cyclosporin A and rituximab and in whom previous genetic testing and a search for multiple infectious causes had been negative. Results: Complete remission of severe nephrotic syndrome was achieved 2 months after the last infusion with, to date, sustained resolution of proteinuria and normal serum albumin levels without further use of IMS drugs and no severe adverse effects noted. Conclusions: Obinutuzumab may be a rescue option for severe, multidrug-resistant ISN, even in young children, when no other therapeutic options are available.

## 1. Introduction

Primary steroid-resistant nephrotic syndrome (SRNS) is an ominous diagnosis in a child with nephrotic syndrome due to limited therapeutic options and high risk of a poor outcome. On recognition, a thorough search for infectious agents, including atypical etiologies, is mandatory, especially in very young children. IPNA and KDIGO recommendations underscore the importance of both early genetic testing and renal biopsy in order to differentiate between a genetic etiology unresponsive to immunosuppressive drugs (IMS) and an immunological disease which may respond to further intensified IMS therapy. If an immunologic background is probable, a course of calcineurin inhibitors (CNIs) and nephroprotective agents is recommended, though the response rate is highly variable 42–85% [1,2,3,4]. Treatment with plasmapheresis or immunoadsorption has been reported in single case reports or small series, with varied outcomes [5,6]. Lately, several reports have been published on the efficacy of rituximab (RTX), an anti-CD20 antibody for SRNS; the presented results have been unequivocal but optimistic, with up to 40.8–58% [7,8] of children achieving remission with different dosing regimens. In SRNS subjects unresponsive to RTX, alternative B cell-depleting agents such as other anti-CD20 agents—obinutuzumab (OBI) and ofatumumab (OFA)—and an anti-CD38 agent, daratumumab (DARA), have been used sporadically [9,10,11,12]. There have been single reports on their successful use in children with difficult-to-treat steroid-dependent nephrotic syndrome (SDNS) [13,14]. Obinutuzumab is a type II anti-CD20 monoclonal antibody which exhibits a longer duration of action than rituximab and induces a deeper, more sustained depletion of lymphocyte B cells. The first and, to date, only report on its use in six children with SRNS was published by Angelettii in 2025 [11]. Anti-CD20 drugs have been predominantly used in children over 7 yrs of age due to their unknown influence on the developing immune system and concern over a potentially greater risk of severe infections in younger age group [15]. The presented report is the second report on the successful use of OBI in a child with primary multidrug-resistant nephrotic syndrome and the first to describe the successful and safe use of this alternate agent in a very young, 2-year-old child.

## 2. Case Presentation

A 19-month-old patient was admitted to a local hospital due to a gastrointestinal infection, presenting with vomiting and diarrhea. Laboratory investigations revealed hypoalbuminemia and nephrotic-range proteinuria. The patient was subsequently transferred to the nearest pediatric nephrology department, where idiopathic nephrotic syndrome (INS) was diagnosed, and standard corticosteroid therapy was started. The child did not respond to 4 weeks of daily standard steroids and therefore methylprednisolone pulses (MP) (15 mg/kg) and cyclosporin were initiated and antiproteinuric therapy with an angiotensin-converting enzyme inhibitor (ACE inhibitor) was continued. A kidney biopsy demonstrated features consistent with focal segmental glomerulosclerosis (FSGS) with diffuse mesangial hypercellularity and no visible immunoglobulins or complement on immunofluorescence. Genetic testing for congenital causes of proteinuria, which included an NGS panel and whole-exome sequencing (WES), yielded negative results. Immunological markers, such as ANA-Hep2, ANCA, and anti- PLA2R antibodies, were negative; C3 and C4 levels were within the normal reference range. Screening for subclinical viral infections excluded an EBV, CMV, HIV, HBV, and HCV etiology. Initial hospitalization was complicated by gastrointestinal infection caused by Clostridium difficile, catheter-related infection, and acute kidney injury (AKI) requiring peritoneal dialysis for 2 weeks. The latter complication was presumably drug-related (due to introduction of cyclosporine) or caused by concomitant severe infection. Due to the severe course of nephrotic syndrome and lack of response to steroids and cyclosporine A (CSA), the boy received two doses of 375 mg/m^2^ BSA of rituximab without any response. Subsequently, further complications developed, including AKI and pronounced edema, necessitating the re-initiation of peritoneal dialysis, which was changed after a few days to continuous renal replacement therapy (CRRT) because of a malfunctioning Tenckhoff catheter. Following four months of hospitalization, during which the boy had received multiple antibiotics, antifungal therapy, and frequent albumin infusions and had required renal replacement therapy, he was transferred to our department for further evaluation and treatment. Renal replacement therapy was continued for the next eleven days. Electrolyte disturbances, oliguria, and edema were gradually corrected, allowing for the discontinuation of albumin infusions. Further rare infections, including HSV-1/2, Candida, Aspergillus, Clostridium, tuberculosis, Pneumocystis, Treponema pallidum, Toxoplasma, rubella, and malaria were excluded. Genetic reanalysis confirmed the previous negative results. The child was classified as having multidrug-resistant INS following previous unsuccessful intensive steroid therapy, CSA, and RTX treatment. Immunophenotyping performed 50 days after the last RTX infusion revealed the presence of, albeit low counts of, CD20/CD19-positive cells (CD20:0.092 g/L and CD19: 0.082 G/L) and switched memory B cells within normal limits (2.8% = 9.0 cells/µL; local laboratory reference range: 1.9–30.4% = 5–74 cells/µL). His serum immunoglobulin levels were within age-related reference values for IgM (0.42 g/L) and IgA (0.44 g/L) but low for IgG (0.48 g/L). Given the high probability of an immunological etiology of the FSGS observed in the boy, the severe, complicated course of his SRNS, and the poor efficacy of previous rituximab therapy, we decided to administer a more potent anti-CD20 drug, obinutuzumab. OBI administration, aimed at achieving a more effective and sustained depletion of B cells, was preceded by a renewed attempt at decreasing his proteinuria with high-dose steroids and intensive symptomatic treatment to minimize drug loss with heavy proteinuria. He received three consecutive daily pulses of MP, followed by weekly infusions for a month. He continued to require regular infusions of 20% albumin to control his edema. Following Bioethical Committee approval for nonstandard treatment of SRNS in a young child (KB/108/2025), OBI was given at a dose of 100 mg (300 mg/1.73 m^2^ BSA) on 13 March 2025 and repeated 2 weeks later. Both infusions were preceded by premedication with antihistamine (cetirizine), acetaminophen, and methylprednisolone (i.v.), as well as montelukast, and were well tolerated. Concomitant cotrimoxazole prophylaxis was prescribed. Prior to the first infusion of obinutuzumab, a single dose of immunoglobulin replacement therapy (IGRT) was administered. Two months after the second infusion, the patient achieved complete remission of proteinuria. Corticosteroid therapy was tapered and finally discontinued, and no other drugs were introduced. To date, the boy has remained in remission. At 7 months following therapy, he continues to demonstrate complete depletion of CD19/CD20 cells and memory B cells. His serum immunoglobulin levels following OBI therapy are below reference values in all three classes. IgG levels have increased to 0.75 g/L in comparison to levels noted prior to obinutuzumab, but IgM and IgA levels have dropped significantly (IgM < 0.1 g/L, IgA − 0.11 g/L). No infection has been noted to date.

## 3. Discussion

We report on the successful and safe use of rescue therapy with obinutuzumab in a very young (2-year-old) child with primary multidrug-resistant nephrotic syndrome in whom other therapies have failed. Standard therapy with steroids, MPs, CSA, and RTX were initially applied, with no improvement over 5 months. Severe complications in the form of massive edema, AKI requiring peritoneal dialysis, and catheter-related infectious complications were initially noted, adding to the risk of an already poor outcome for the clinical diagnosis of primary SRNS. Renal biopsy revealed focal segmental glomerulosclerosis, a further poor prognostic factor for ISN with a steroid-resistant course. Extensive genetic analysis (NGS and WES) did not reveal any pathogenic variants for a genetic pathogenesis of the observed SRNS. A thorough search for ongoing causative infectious agents which may be associated with INS, especially in the youngest age group, was also negative. A diagnosis of a probably immunological multidrug-resistant INS was made in the 2-year-old subject. The therapeutic options for such a diagnosis are extremely restricted [1,4,6,16,17], and the risk of a rapid progression to end-stage renal disease is high. Following the efficacy of anti-CD20 drugs for the treatment of complicated SDNS/FRNS, several reports have been published on their use in SRNS [7,8]. The most frequently used agent has been rituximab. The response rates of children with SRNS to RTX have been variable, from 30% to even 80%, suggesting that this group of drugs may be an option for children with immunological ISN resistant to standard therapy. The data are nevertheless confusing due to the diversity of patients described, including subjects with both primary and secondary steroid resistance. Several single case publications have reported on the successful use of RTX in SRNS children [18,19,20]. However, multicenter studies have reported different efficacy rates for RTX therapy [7,21,22,23,24]. Furthermore, the only randomized controlled trial evaluating the efficacy of rituximab as add-on therapy in 31 patients resistant to steroids and cyclosporin showed no benefit after 3 months of treatment [25]. Factors for better response to RTX therapy in SRNS have included minimal change disease in comparison to focal segmental glomerulosclerosis on biopsy [8,23]. A Japanese multicenter retrospective study of 45 SRNS children concluded that early treatment (before 6 months from onset) with RTX, together with high-dose steroids and IMS agents, was associated with a complete response [7]. On the other hand, steroid resistance occurring at a very young age may be the result of an underlying genetic podocytopathy with a low likelihood of response to intensified immunosuppression [22]. Our patient was younger than 2 years old at the moment of diagnosis, and genetic analysis was mandatory before any further IMS therapy was commenced. The negative result of a comprehensive NGS panel and further WES study suggested a high probability of an immunological background of the disease. We were not able at the time to assess anti-nephrin antibodies in the boy, but immunophenotyping revealed that 5 weeks following two RTX infusions, he was already repopulating B cells and his memory B cells were intact. This could be due to underdosing of RTX observed in children with active NS and severe protein loss. The optimal dosing of RTX for both SDNS and SRNS is still under debate [4,16,17]. Drug loss due to heavy proteinuria may lead to decreased serum drug availability and efficacy; higher initial doses of RTX have been suggested by some authors to have a beneficial effect on achieving remission [24]. There are single reports on the shorter half-life of RTX in children with heavy proteinuria with an even twentyfold shorter half-life of the drug reported due to urinary loss of anti-CD20 antibody [8,26]. Our patient had initially received two consecutive infusions of 375 mg/m^2^ BSA of RTX and depletion of CD20 cells had been achieved but was short-lasting (5 weeks). We decided to use a drug that seems to have a more potent anti-CD20 effect. There have been single publications on the use of obinutuzumab and ofatumumab in children with INS whose response to RTX had not been satisfactory. Ofatumumab therapy has been used with success in children with steroid-resistant INS. OFA is a human monoclonal antibody which differs from obinutuzumab in its mechanism of action. The drug induces stronger complement-dependent cytotoxicity (CDC) but weaker antibody-dependent cellular cytotoxicity (ADCC)**.** Several publications have described the successful use of ofatumumab in children with complicated nephrotic syndrome [9,10]. Two reports of ofatumumab therapy in children with SRNS (5–19 yrs age) have shown encouraging results of complete remission [9,10]. However, the single randomized trial with use of OFA in children with SDNS, performed by Ravani et al., did not show superiority of ofatumumab over RTX therapy in this group of patients [27]. Daratumumab is an anti-CD38 antibody. CD38 is present on different subtypes of B cells, including plasma cells. Therefore, DARA is regarded as a more potent B cells-depleting agent in comparison to other anti-CD20 drugs. Successful use of combined anti-CD20 and anti-CD38 therapy in children with complicated or resistant nephrotic syndrome has been reported [12,28]. When looking for an alternative anti-CD20 agent for our SRNS subject, the only available option was Obinutuzumab, as Ofatumumab had been withdrawn from the European market by its producer, and the availability of daratumumab was very limited [29]. Obinutuzumab has been used with success in patients with severe or complicated steroid-dependent nephrotic syndrome [13,14,30]. The first and single report on its use in SRNS comes from Angeletti et al. [11]. Three of the six pediatric SRNS subjects (all older than 7 years of age) achieved complete remission of nephrotic syndrome. The recommended dose of obinutuzumab in children with leukemia is 1000 mg/m^2^ BSA, or 15 mg/kg in children with a smaller body weight. In a study by Dossier et al. reporting the use of the drug in children with steroid-dependent nephrotic syndrome, lower doses (300 mg/1.73 m^2^) were used successfully [13]. Due to the young age of our patient, we decided to prescribe a lower dose but to administer two consecutive doses to compensate for the predicted loss due to massive proteinuria. There had been no published data on pharmacokinetics of obinutuzumab in patients with heavy proteinuria to guide our dosing. Currently, two clinical trials assessing the use of OBI in children with INS and lupus nephritis are ongoing (NCT05627557, NCT05039619), and their results may be able to address this important issue [31,32]. The issue of altered pharmacokinetics of anti-CD20 drugs in patients with heavy proteinuria and their necessary dose adjustments in this patient population has been raised [26,33].

In spite of the limited data available, the preliminary findings of the mentioned studies suggest that alternate anti-CD20 therapies may be a potential therapeutic option in pediatric SRNS patients resistant to RTX in whom other therapeutic options have expired. The successful use of obinutuzumab in the presented case report of a boy with multidrug-resistant INS confirms the initial report of Angeletti on its potentially effective use for this indication. An important consideration in the use of this drug is the subject’s age. Young age and preceding hypogammaglobulinemia have been looked upon as potential risk factors for complications of anti-CD20 therapy, severe infections being the major concern. The 2023 IPNA guidelines recommend administration of these agents in older children, typically over 7 years of age [4,16]. Nevertheless, SRNS due to INS is frequently diagnosed in young children, and multidrug-resistant nephrotic syndrome is frequently accompanied by hypogammaglobulinemia due to massive proteinuria which includes IgG loss. To reduce the risk of infectious complications associated with the observed hypogammaglobulinemia in our patient, we decided to administer immunoglobulin replacement therapy prior to starting OBI treatment. He received only a single dose, as he improved rapidly, and his IgG levels increased when he achieved remission.

This case report underscores the successful use of obinutuzumab in a very young 24-month-old child with a 5-month history of severe complicated primary SRNS refractory to steroids, CSA, and RTX with underlying FSGS histopathology, further increasing the child’s risk of a poor renal outcome. It highlights the challenges of using anti-CD20 therapies in this age group but demonstrates the potential applicability of obinutuzumab even in very young patients. The administration of obinutuzumab allowed the patient to overcome not only steroid and CSA resistance but also the initial resistance to rituximab. The child had required renal replacement therapy on two occasions and had succumbed to many infections during his unresolved severe nephrotic syndrome. Early treatment with OBI facilitated both the discontinuation of further previously unsuccessful trials of intensified steroids and immunosuppressive drugs and the change from a dismal prognosis of multidrug-resistant INS to a more hopeful OBI-sensitive INS, though the duration of sustained disease remission is unknown and future treatment decisions in case of a future relapse remain debatable.

## 4. Conclusions

Obinutuzumab may be considered a rescue therapy for severe multidrug-resistant INS, even in young children, when other therapeutic options have expired.

## Data Availability

Data are unavailable due to privacy and ethical restrictions.

## Abbreviations

The following abbreviations are used in this manuscript:

SRNS	steroid-resistant nephrotic syndrome
IMS	immunosuppressive drugs
CNI	calcineurin inhibitors
RTX	rituximab
OBI	obinutuzumab
OFA	ofatumumab
SDNS	Steroid-dependent nephrotic syndrome
INS	idiopathic nephrotic syndrome
MP	methylprednisolone pulse
FSGS	focal segmental glomerulosclerosis
ACE	angiotensin-converting enzyme
WES	whole-exome sequencing
AKI	acute kidney injury
CSA	cyclosporine A
IGRT	immunoglobulin replacement therapy
